# COI barcoding of plant bugs (Insecta: Hemiptera: Miridae)

**DOI:** 10.7717/peerj.6070

**Published:** 2018-12-04

**Authors:** Junggon Kim, Sunghoon Jung

**Affiliations:** Laboratory of Systematic Entomology, Department of Applied Biology, College of Agriculture and Life Sciences, Chungnam National University, Daejeon, Korea

**Keywords:** DNA barcoding, COI, Insects, Plant bugs, Miridae

## Abstract

The family Miridae is the most diverse and one of the most economically important groups in Heteroptera. However, identification of mirid species on the basis of morphology is difficult and time-consuming. In the present study, we evaluated the effectiveness of COI barcoding for 123 species of plant bugs in seven subfamilies. With the exception of three *Apolygus* species—*A. lucorum*, *A. spinolae*, and *A. watajii* (subfamily Mirinae)—each of the investigated species possessed a unique COI sequence. The average minimum interspecific genetic distance of congeners was approximately 37 times higher than the average maximum intraspecific genetic distance, indicating a significant barcoding gap. Despite having distinct morphological characters, *A. lucorum*, *A. spinolae*, and *A. watajii* mixed and clustered together, suggesting taxonomic revision. Our findings indicate that COI barcoding represents a valuable identification tool for Miridae and can be economically viable in a variety of scientific research fields.

## Introduction

Heteroptera (Insecta: Hemiptera)—commonly termed true bugs—comprises the largest global group of hemimetabolous insects, having more than 42,000 described species in 5,800 genera and 140 families (Henry, 2009). The family Miridae (plant bugs) represents the largest and one of the most economically important heteropteran groups. This group contains many well-known insect pests such as alfalfa bugs (*Adelphocoris lineolatus*) and tarnished plant bugs (*Lygus rugulipennis*), as well as predators that can be used as biological control agents (e.g., *Nesidiocoris tenuis* and *Cyrtorhinus lividipennis*) (Schaefer & Panizzi, 2000; Wheeler, 2001). A pre-requisite for control and/or application is reliable identification. However, identification of mirid species on the basis of morphological characters is difficult and time-consuming (Raupach et al., 2014).

DNA barcoding using partial DNA sequences such as mitochondrial cytochrome c oxidase subunit I (COI) is a valuable tool for identifying and distinguishing between species in various animal taxa (e.g., birds, fishes, and insects) (Hebert et al., 2004a; Hebert et al., 2004b; Ward et al., 2005; Yoo et al., 2006; Foottit et al., 2008; Jung, Duwal & Lee, 2011). To evaluate effectiveness of this method, the average intraspecific and the average interspecific genetic distance are investigated. Additionally, ‘barcoding gap’, a significant difference between intraspecific and interspecific genetic distance is detected. This gap is considered to have a difference with at least 10 times higher average interspecific distance than average intraspecific distance (Candek & Kuntner, 2015). This approach can also be used to discover hidden and/or new species and to identify morphologically cryptic species (Hebert et al., 2004a; Hebert et al., 2004b; Jung, Duwal & Lee, 2011). However, no study evaluates the utilities of barcoding in the family Miridae, Furthermore, there are few available barcode data for identification of this group, given high species diversity and morphological similarity. The objective of the present study was to evaluate the efficiency of COI barcoding as an identification tool for Miridae, and to obtain COI barcoding data for 274 individuals belonging to 123 species in this family.

## Materials and Methods

With the exception of the rare group Psallopinae, we sampled species belonging to all mirid subfamilies: Bryocorinae, Cylapinae, Deraeocorinae, Isometopinae, Mirinae, Orthotylinae, and Phylinae. Detailed information (e.g., collection data, collector, collection locations and coordinates, GenBank accession number) is presented in Table S1. An average of 2.2 specimens per species was used in this study. The obtained samples were preserved in absolute ethanol. Prior to DNA extraction, we performed morphological identification on the basis of genitalia structure with the published literatures (e.g., Josifov, 1976; Kerzhner, 1988; Yasunaga, 1991; Yasunaga, 1999; Duwal et al., 2012; Kim & Jung, 2015; Kim & Jung, 2016a; Kim & Jung, 2016b; Kim & Jung, 2016c). The remaining parts after morphological identification were deposited as voucher specimens in the Laboratory of Systematic Entomology, Chungnam National University (CNU), Daejeon, Korea.

Genomic DNA was extracted either from whole samples or from the remaining tissues after morphological identification using a QIAamp DNA Mini Kit in accordance with the manufacturer’s protocol (Qiagen, Hilden, Germany). PCR was performed using the Solg 2X Taq PCR Pre-mix (SolGent, Daejeon, South Korea) with the primer pair LCO1490 and HCO 2190 (Folmer et al., 1994). The thermal cycling program comprised an initial step of 95 °C for 2 min; 35 cycles each of 95 °C for 20 s, 45–48 °C for 40 s, and 72 °C for 1 min; and a final extension step of 72 °C for 5 min. The PCR products were purified using an MG™ PCR SV purification kit (MGmed Inc.) and sequenced using a 96-capillary ABI PRISM 3730xl DNA analyzer (Macrogen, Seoul, South Korea). The obtained sequences were aligned using Megalign (DNA-star™) and MEGA version 5.2 (Tamura et al., 2011); none of these sequences was found to possess indels. Sequence divergences were calculated using the Kimura-2-parameter model (K2P) (Kimura, 1980), and the trees were generated using the neighbor-joining method (NJ) (Saitou & Nei, 1987), followed as one of the general protocols for barcoding study. The barcoding gap was investigated by calculating the average of maximum intraspecific distance of individuals in each species, and the average of minimum interspecific genetic distance between congeners to make it minimal and strict. To investigate general intraspecific and interspecific distance in each taxonomic level, each value was calculated using all individuals of each species for intraspecific distance, and calculated using all species in same genus, respectively. All sequences obtained in this study were deposited in NCBI (GenBank accession numbers KY366988–KY367257 and KY229058, KY229059, KY229060, KY229061, corresponding to the voucher numbers presented in Table S1).

## Result

**Figure 1:**
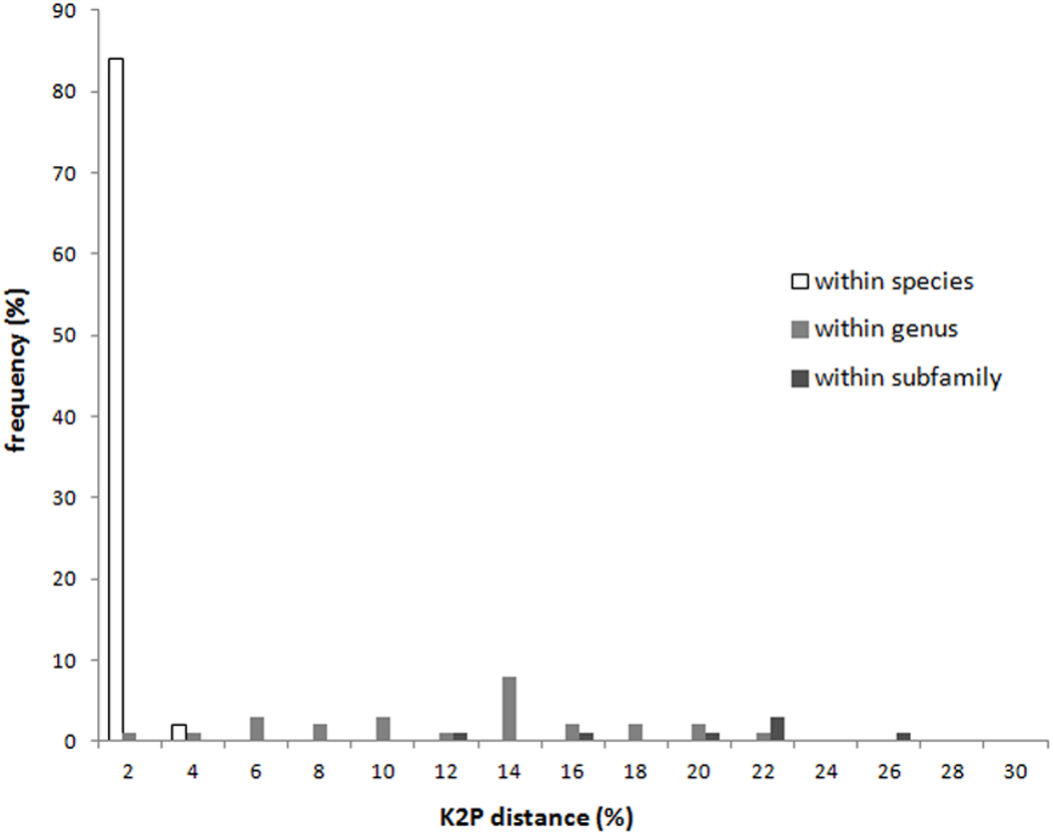
Intraspecific distance and interspecific distances within genus and subfamily of COI sequences for each taxonomic level of Miridae.

With the exception of *Apolygus lucorum*, *A. spinolae*, and *A. watajii*, each of the investigated species possessed a unique COI sequence (Fig. S1). The K2P distances of the COI regions of specimens at each taxonomic level are shown in Fig. 1 and Table 1. The intraspecific sequences from individuals of 57 species were either identical or very similar. The average minimum interspecific genetic distance between congeners (11.2%) was about 37 times higher than the average maximum intraspecific genetic distance (0.3%), indicating a significant barcoding gap. The maximum intraspecific genetic distance exceeded 2% in two species—*Apolygus watajii* (2.6%) and *Eurystylus coelestialium* (2.8%).

**Table 1:** K2P sequence distances and comparisons to previous studies for heteropteran species at each taxonomic levels. Corresponding values from previous studies are given in parentheses, respectively.

	**Range (%)**		**Mean (%)**
Intraspecific distances	0–2.8 (N/A^a^, 0–7.72^b^, 0–23.31^c^)		0.2 (0.8^a^, 0.74^b^, 0.45^c^)
Interspecific distances of congeners	0–20.4 (N/A^a^, 0–24.8^b^, 0–27.67^c^)		11.36 (12.6^a^, 10.67^b^, 13.59^c^)
Interspecific distance in subfamily (N/A^a,b,c^)	Bryocorinae	19.4–29.3	18.87
	Cylapinae	20.6–20.9	
	Deraeocorinae	12.7–25.7	
	Isometopinae	1.8–16.0	
	Mirinae	0–24.1	
	Orthotylinae	1.6–27.5	
	Phylinae	7.1–26.4	

**Notes.**

a Jung, Duwal & Lee (2011).

b Park et al. (2011).

c Raupach et al. (2014).

**Figure 2:**
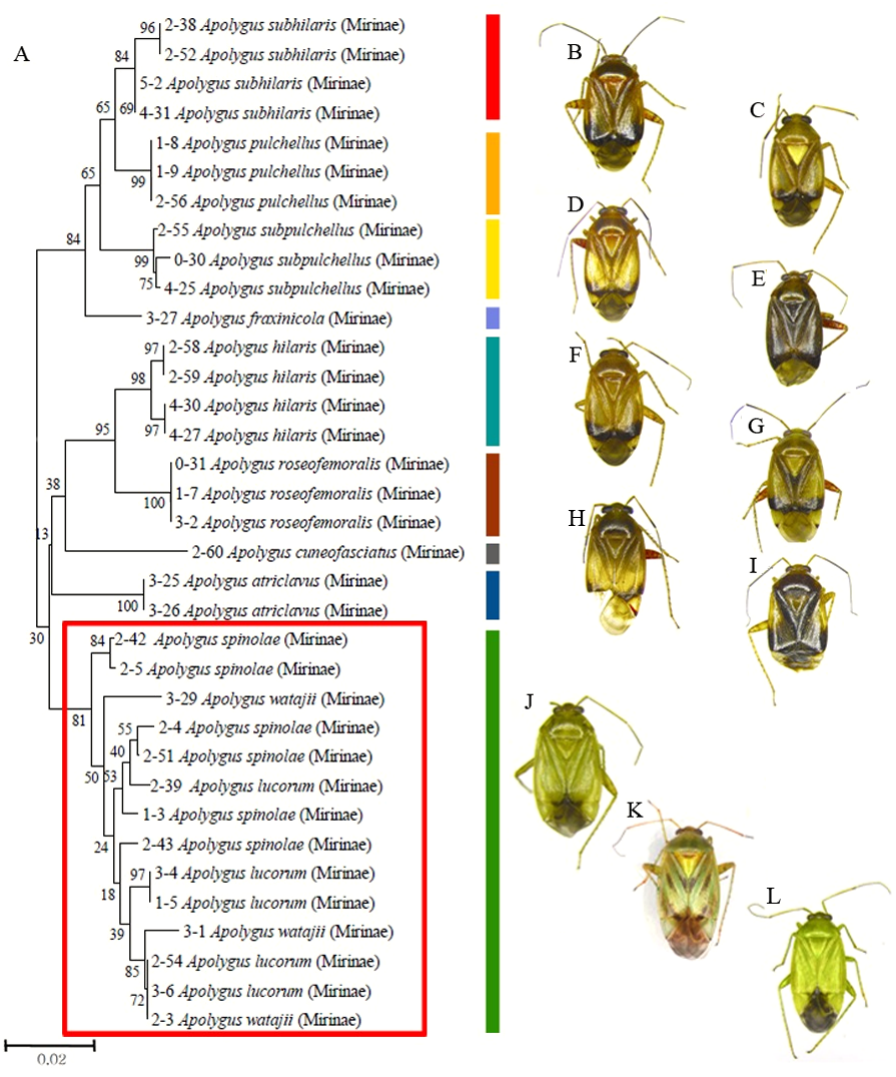
Partial neighbor-joining tree and dorsal habitus of *Apolygus* species. (A) Neighbor-joining tree of 35 COI sequences from 11 *Apolygus* species; (B–L) Dorsal habitus of 11 species; (B) *A. subhilaris*; (C) *A. pulchellus*; (D) *A. subpulchellus*; (E) *A. fraxinicola*; (F) *A. hilaris*; (G) *A. roseofemoralis*; (H) *A. cuneofasciatus*; (I) *A. ctriclavus*; (J) *A. lucorum*; (K) *A. spinolae*; (L) *A. watajii*; red box with green bar indicates three mixed greenish species (J–L).

Of the 11 investigated *Apolygus* species, three greenish species—*A. lucorum*, *A. spinolae*, and *A.watajii*—mixed and clustered together (Fig. 2; red box). For these three species, the average interspecific genetic distance (1.4%) was much lower than the average interspecific genetic distance for other species in the same genus (not greenish; 4.4%). In addition, the average intraspecific genetic distance for these three species was markedly higher (1.7%) than the average intraspecific genetic distance for other species in this study (0.2%).

With the exception of some splitting of genera and subfamilies, the tree constructed using the COI sequences was in good agreement with the taxonomic classification based on morphological characters, from species level to subfamily level (Fig. S1). The interspecific genetic distance of each subfamily was shown in Table 1. The average intraspecific genetic distance was 0.2%; the average interspecific genetic distance for congeneric species was 11.36%. Additionally, the average interspecific distance for species within the same subfamily was 18.87% (Table 1).

## Discussion

In the present study, we investigated the barcoding gap and evaluated the effectiveness of COI barcoding for Miridae, by determining the level of intraspecific variation. We found that the mean and range of the average maximum intraspecific genetic distance overlapped with those obtained in previous studies of hemipteran and mirid species (Jung, Duwal & Lee, 2011; Park et al., 2011; Raupach et al., 2014; Tembe, Shouche & Ghate, 2014). Comparison of the average minimum interspecific genetic distance for congeners with the maximum intraspecific divergence within each species yielded similar results to those obtained in previous studies of other heteropteran groups (Jung, Duwal & Lee, 2011; Park et al., 2011; Raupach et al., 2014; Tembe, Shouche & Ghate, 2014). Thus, we verified the usefulness of DNA barcoding for the plants bugs investigated in the present study.

We calculated intraspecific variations using the limited samples collected from the same region (only collected in Korea; Table S1). Our results based on sampling from the same geographic area showed intraspecific genetic distances comparable to previous reports that used extensive sampling of Miridae and from a larger geographic region (Park et al., 2011) (Table 1). However, no meaningful differences related to intraspecific variations were observed when compared to previous studies conducted with a relatively larger sample size from various regions and/or countries (Jung, Duwal & Lee, 2011; Park et al., 2011; Raupach et al., 2014; Tembe, Shouche & Ghate, 2014).

**Figure 3:**
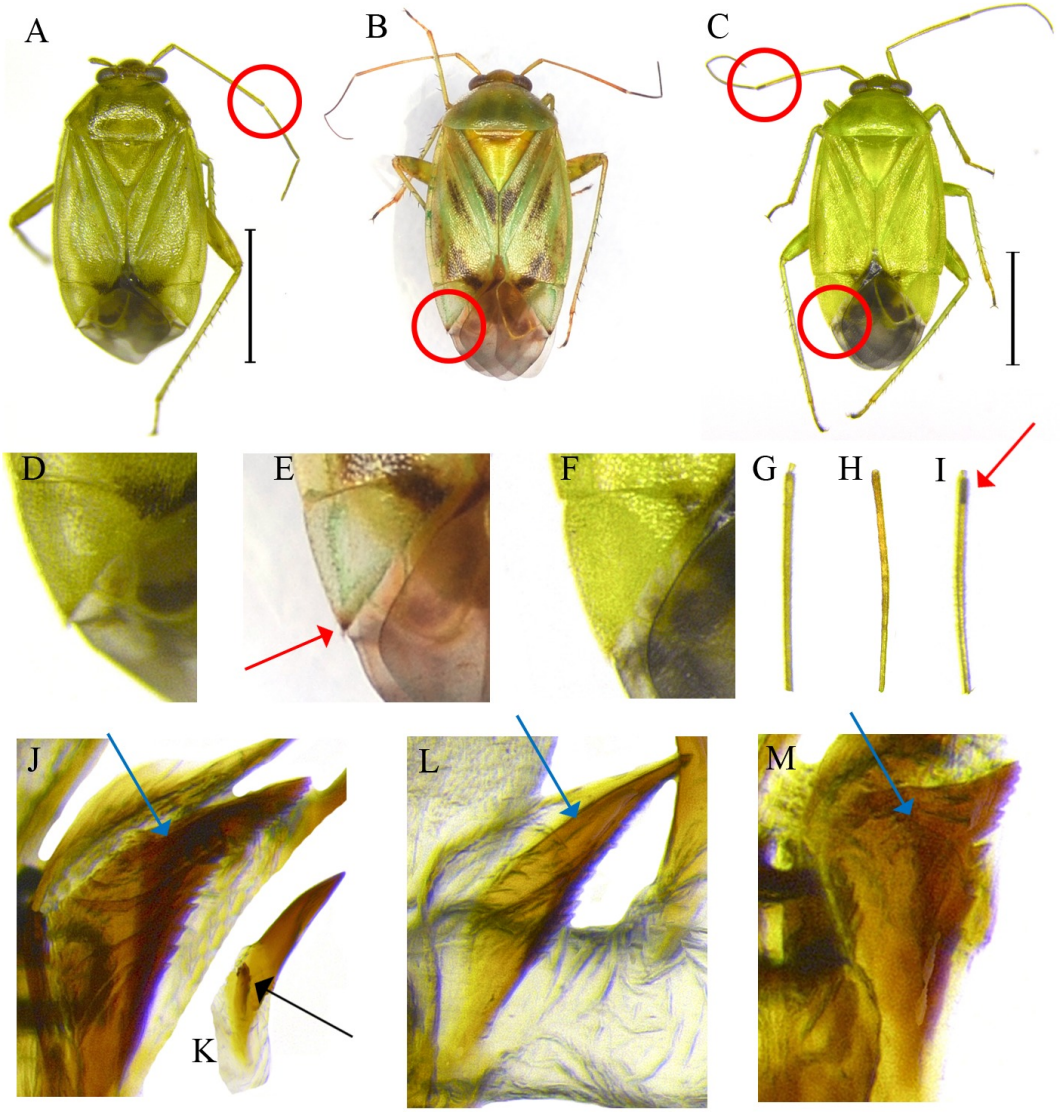
External and genital structures as diagnostic characters of three *Apolygus* species. (A, D, G, J, K) *A. lucorum*; (B, E, H, L) *A. spinolae*; (C, F, I, M) *A*. *watajii*; (A–C) dorsal habitus of adult; (D–F) apex of cuneus; (G–I) apex of 2nd antennal segment; (J–M) structures of endosoma; red circles, dark markings of 2nd antennal segment and cuneus; red arrows, magnified characters of red circles in figures; blue arrows, structures of wing-shaped sclerites; black arrow, sublateral sclerite, respectively.

Three greenish species in the genus *Apolygus* (*A. lucorum*, *A. spinolae*, and *A. watajii*) clustered separately from other congeneric species, and mixed clades of these three species were indicated (Fig. 2). This finding was in agreement with that of Jung, Duwal & Lee (2011), despite the fact that we investigated an additional six species in our present study. *A. lucorum*, *A. spinolae*, and *A. watajii* can be distinguished on the basis of the following morphological characters: coloration of apex of second antennal segment; presence or absence of dark marking of apex of cuneus; structure of wing-shaped sclerite (*ws*) and presence or absence of sublateral sclerite (*sls*) of endosoma (*Apolygus lucorum*—*ws* broad and dentate laterally, *sls* present; *Apolygus spinolae*—*ws* slender, *sls* absent; *Apolygus watajii*—*ws* distinctly short and broad with lateral dentate, *sls* absent and ventral sclerite (*vsc*) extremely sharp and long (Yasunaga, 1991; Yasunaga & Yasunaga, 2000)) (Fig. 3). Nevertheless, in the present study, we were unable to identify these three species on the basis of COI sequences. Yang et al. (2015) suggested that different pheromone components and ratios in *A. lucorum* and *A. spinolae* females play important roles in reproductive isolation. These two species were subsequently distinguished on the basis of mitochondrial COI and 16S rRNA sequences (Yang et al., 2016). However, a total of six nucleotides were different in the sequences with 709 bp in COI between two species, which means that the genetic distance between two species is less than 1%. Therefore, further studies using additional species in the genus *Apolygus*—especially greenish species—and based on different morphological data, and molecular markers are required to clarify the taxonomies of these species.

## Conclusion

Most mirids are economically important insect pests or biological control agents in the agricultural and forestry sector, and also play key roles in the ecosystem (Wheeler, 2000a; Wheeler, 2000b; Wheeler, 2001). However, the molecular resources less than 3.5% of described species have been constructed for identification and application. The objective of this study were to evaluate the usefulness of COI barcoding for Miridae, to construct COI barcode sequence data based on morphologically identified species by authors (e.g., Jung et al., 2010; Duwal et al., 2012; Jung, Duwal & Lee, 2012; Duwal, Jung & Lee, 2013; Duwal, Jung & Lee, 2014; Kim et al., 2015; Kim & Jung, 2015; Kim & Jung, 2016a; Kim & Jung, 2016b; Kim & Jung, 2016c; Kim & Jung, 2017) and finally to provide reliable molecular resources for various researchers. As a result, all the mirids in this study could be identified using the COI barcode, except for the green *Apolygus* species. We propose that COI barcoding represents a valuable identification tool for Miridae and may be economically viable in a variety of scientific applications.

##  Supplemental Information

10.7717/peerj.6070/supp-1 Table S1 274 Samples used in this study Abbreviations: CNU, Chungnam National University; CB, Chungcheongbuk-do; CN, Chungcheongnam-do; GB, Gyeongsangbuk-do; GG, Gyeonggi-do; GN, Gyeongsangnam-do; GW, Gangwon-do; JB, Jeollabuk-do; JN, Jeollanam-do; JJ, Jeju-do.

10.7717/peerj.6070/supp-2 Supplemental Information 1 COI sequence alignment used in this study

10.7717/peerj.6070/supp-3 Figure S1 Neighbor-Joining tree of 274 COI sequences from Plant Bugs Parenthesis indicates the subfamily of the species. Numbers indicates specimen numbers corresponding to Table S1.

## Additional Information and Declarations

### Data Availability

The following information was supplied regarding data availability: All sequences obtained in this study were deposited in NCBI (GenBank accession numbers KY366988–KY367257 and KY229058, KY229059, KY229060, KY229061). The accession number of raw data is in Table S1.
