# Supplementary material for: COI barcoding of plant bugs (Insecta: Hemiptera: Miridae)
Source: PeerJ. 2018 Dec 4;6:e6070. doi: 10.7717/peerj.6070 (PMC6284446; doi:10.7717/peerj.6070)
Supplement: Table S1 — Abbreviations: CNU, Chungnam National University; CB, Chungcheongbuk-do; CN, Chungcheongnam-do; GB, Gyeongsangbuk-do; GG, Gyeonggi-do; GN, Gyeongsangnam-do; GW, Gangwon-do; JB, Jeollabuk-do; JN, Jeollanam-do; JJ, Jeju-do. [file peerj-06-6070-s001.docx]

**SI Table. 274 Samples used in this study.** Abbreviations: CNU, Chungnam National University; CB, Chungcheongbuk-do; CN, Chungcheongnam-do; GB, Gyeongsangbuk-do; GG, Gyeonggi-do; GN, Gyeongsangnam-do; GW, Gangwon-do; JB, Jeollabuk-do; JN, Jeollanam-do; JJ, Jeju-do.

| Specimen number | Subfamily | Genus | Species | Collecting site  (Coordinate) | Collecting date  (date-month-year) | Voucher number | GenBank  Accession numbers |
| --- | --- | --- | --- | --- | --- | --- | --- |
| 3-58 | Bryocorinae | *Bryocoris* | *gracilis* | Korea, JB  35.32N 126.66E | 31-Jul-14 | CNUHHM025022 | KY366988 |
| 0-7 | Bryocorinae | *Cyrtopeltis* | *miyamotoi* | Korea, JJ  33.43N 126.62E | 19-Jun-14 | CNUHHM040025 | KY366989 |
| 0-8 | Bryocorinae | *Cyrtopeltis* | *miyamotoi* | Korea, CN  34.35N 126.66E | 05-Jun-14 | CNUHHM013087 | KY366990 |
| 0-9 | Bryocorinae | *Cyrtopeltis* | *rufobrunnea* | Korea, GW  37.71N 128.71E | 11-Jul-14 | CNUHHM020072 | KY366991 |
| 3-37 | Bryocorinae | *Cyrtopeltis* | *rufobrunnea* | Korea, JB  35.32N 126.66E | 31-Jul-14 | CNUHHM025030 | KY366992 |
| 5-44 | Bryocorinae | *Cyrtopeltis* | *rufobrunnea* | Korea, GW  37.71N 128.71E | 17-Jul-15 | CNUHHM037018 | KY366993 |
| 3-56 | Bryocorinae | *Michailocoris* | *josifovi* | Korea, CN  36.37N 127.35E | 12-Aug-15 | CNUHHM033061 | KY366994 |
| 3-57 | Bryocorinae | *Michailocoris* | *josifovi* | Korea, CN  36.37N 127.35E | 28-Jul-14 | CNUHHM015028 | KY366995 |
| 0-10 | Bryocorinae | *Nesidiocoris* | *tenuis* | Korea, CN  36.37N 127.35E | 11-Aug-14 | CNUHHM009079 | KY366996 |
| 0-11 | Bryocorinae | *Nesidiocoris* | *tenuis* | Korea, CN  36.37N 127.35E | 22-Aug-14 | CNUHHM014009 | KY366997 |
| 0-12 | Bryocorinae | *Nesidiocoris* | *tenuis* | Korea, JN  34.35N 126.66E | 16-Jun-14 | CNUHHM009050 | KY366998 |
| 4-38 | Cylapinae | *Fulvius* | *anthocoroides* | Korea, CN  36.37N 127.35E | 28-Jul-14 | CNUHHM015027 | KY366999 |
| 5-60 | Cylapinae | *Fulvius* | *anthocoroides* | Korea, GW  37.71N 128.71E | 10-Jul-14 | CNUHHM023079 | KY367000 |
| 4-37 | Cylapinae | *Punctifulvius* | *kerzhneri* | Korea, JJ  33.43N 126.62E | 03-Sep-15 | CNUHHM036013 | KY367001 |
| 5-12 | Cylapinae | *Punctifulvius* | *kerzhneri* | Korea, GW  37.71N 128.71E | 18-Aug-16 | CNUHHM000058 | KY367002 |
| 3-52 | Deraeocorinae | *Alloeotomus* | *chinensis* | Korea, CN  36.37N 127.35E | 14-Jul-14 | CNUHHM040050 | KY367003 |
| 5-55 | Deraeocorinae | *Alloeotomus* | *chinensis* | Korea, GW  37.71N 128.71E | 10-Jul-14 | CNUHHM017086 | KY367004 |
| 5-56 | Deraeocorinae | *Alloeotomus* | *chinensis* | Korea, CB  36.37N 127.35E | 06-Aug-15 | CNUHHM033049 | KY367005 |
| 0-43 | Deraeocorinae | *Alloeotomus* | *simplus* | Korea, JN  34.35N 126.66E | 25-Jul-16 | CNUHHM043032 | KY367006 |
| 3-51 | Deraeocorinae | *Alloeotomus* | *simplus* | Korea, CN  36.37N 127.35E | 31-Jul-15 | CNUHHM038082 | KY367007 |
| 0-72 | Deraeocorinae | *Bothynotus* | *pilosus* | Korea, CN  36.37N 127.35E | 13-Jun-14 | CNUHHM011085 | KY367008 |
| 0-67 | Deraeocorinae | *Cimidaeorus* | *hasegawai* | Korea, GG  37.21N 126.64E | 24-May-14 | CNUHHM000006 | KY367009 |
| 0-39 | Deraeocorinae | *Deraeocoris* | *ater* | Korea, GW  37.71N 128.71E | 09-Jul-14 | CNUHHM025059 | KY367010 |
| 5-51 | Deraeocorinae | *Deraeocoris* | *ater* | Korea, CN  36.37N 127.35E | 11-Jun-14 | CNUHHM021076 | KY367011 |
| 0-37 | Deraeocorinae | *Deraeocoris* | *castaneae* | Korea, CN  36.37N 127.35E | 28-Jul-14 | CNUHHM015033 | KY367012 |
| 3-41 | Deraeocorinae | *Deraeocoris* | *castaneae* | Korea, CN  36.37N 127.35E | 12-Sep-12 | CNUHHM009032 | KY367013 |
| 3-54 | Deraeocorinae | *Deraeocoris* | *castaneae* | Korea, JB  35.32N 126.66E | 04-Aug-14 | CNUHHM036058 | KY367014 |
| 0-40 | Deraeocorinae | *Deraeocoris* | *claspericapilatus* | Korea, GN  34.47N 128.69E | 23-Jul-15 | CNUHHM040057 | KY367015 |
| 3-48 | Deraeocorinae | *Deraeocoris* | *elegantulus* | Korea, GW  37.71N 128.71E | 06-Jun-15 | CNUHHM000049 | KY367016 |
| 5-49 | Deraeocorinae | *Deraeocoris* | *elegantulus* | Korea, GW  37.71N 128.71E | 29-May-15 | CNUHHM030022 | KY367017 |
| 0-38 | Deraeocorinae | *Deraeocoris* | *olivaceus* | Korea, GW  37.71N 128.71E | 30-May-14 | CNUHHM000002 | KY367018 |
| 5-50 | Deraeocorinae | *Deraeocoris* | *olivaceus* | Korea, GW  37.71N 128.71E | 30-Jun-16 | CNUHHM000067 | KY367019 |
| 0-36 | Deraeocorinae | *Deraeocoris* | *pulchellus* | Korea, GN  34.47N 128.69E | 23-Jul-14 | CNUHHM036051 | KY367020 |
| 3-46 | Deraeocorinae | *Deraeocoris* | *pulchellus* | Korea, CN  36.37N 127.35E | 04-May-15 | CNUHHM027025 | KY367021 |
| 3-47 | Deraeocorinae | *Deraeocoris* | *pulchellus* | Korea, CN  36.37N 127.35E | 18-Jul-15 | CNUHHM040052 | KY367022 |
| 3-39 | Deraeocorinae | *Deraeocoris* | *sanghonami* | Korea, GW  37.71N 128.71E | 28-May-15 | CNUHHM030006 | KY367023 |
| 5-52 | Deraeocorinae | *Deraeocoris* | *sanghonami* | Korea, GW  37.71N 128.71E | 06-Jun-15 | CNUHHM030080 | KY367024 |
| 0-41 | Deraeocorinae | *Deraeocoris* | *ulmi* | Korea, GG  37.21N 126.64E | 15-Feb-14 | CNUHHM017004 | KY367025 |
| 0-42 | Deraeocorinae | *Deraeocoris* | *ulmi* | Korea, CN  36.37N 127.35E | 15-Jun-15 | CNUHHM031060 | KY367026 |
| 5-53 | Deraeocorinae | *Deraeocoris* | *ulmi* | Korea, GG  37.21N 126.64E | 03-Jul-14 | CNUHHM039050 | KY367027 |
| 3-40 | Deraeocorinae | *Deraeocoris* | *yasunagai* | Korea, JB  35.32N 126.66E | 31-Jul-14 | CNUHHM025033 | KY367028 |
| 5-54 | Deraeocorinae | *Deraeocoris* | *yasunagai* | Korea, JN  34.35N 126.66E | 25-Jul-16 | CNUHHM043033 | KY367029 |
| 3-50 | Deraeocorinae | *Stethoconus* | *japonicus* | Korea, GG  37.21N 126.64E | 03-Jul-14 | CNUHHM039049 | KY367030 |
| 5-57 | Deraeocorinae | *Stethoconus* | *japonicus* | Korea, CN  36.37N 127.35E | 31-Jul-15 | CNUHHM039020 | KY367031 |
| 5-48 | Deraeocorinae | *Termatophylum* | *hikosanum* | Korea, JN  34.35N 126.66E | 25-Jul-16 | CNUHHM043013 | KY367032 |
| 4-43 | Isometopinae | *Isometopus* | *amurensis* | Korea, GG  37.21N 126.64E | 18-Jun-16 | CNUHHM000053 | KY367033 |
| 4-44 | Isometopinae | *Isometopus* | *amurensis* | Korea, GG  37.21N 126.64E | 18-Jun-16 | CNUHHM000054 | KY367034 |
| 4-41 | Isometopinae | *Isometopus* | *japonicus* | Korea, JB  35.32N 126.66E | 24-May-15 | CNUHHM034062 | KY367035 |
| 4-42 | Isometopinae | *Isometopus* | *japonicus* | Korea, JB  35.32N 126.66E | 24-May-15 | CNUHHM034063 | KY367036 |
| 3-60 | Isometopinae | *Isometopus* | *jejuensis* | Korea, JJ  33.43N 126.62E | 02-Sep-15 | CNUHHM035055 | KY367037 |
| 1-24 | Mirinae | *Adelphocoris* | *demissus* | Korea, GW  37.71N 128.71E | 16-Sep-15 | CNUHHM000010 | KY367038 |
| 1-16 | Mirinae | *Adelphocoris* | *lineolatus* | Korea, GW  37.71N 128.71E | 16-Sep-15 | CNUHHM000009 | KY367039 |
| 3-11 | Mirinae | *Adelphocoris* | *lineolatus* | Korea, GW  37.71N 128.71E | 29-Jun-16 | CNUHHM042071 | KY367040 |
| 1-50 | Mirinae | *Adelphocoris* | *piceosetosus* | Korea, GB  35.87N 128.35E | 19-Aug-15 | CNUHHM041045 | KY367041 |
| 1-51 | Mirinae | *Adelphocoris* | *piceosetosus* | Korea, GB  35.87N 128.35E | 19-Aug-15 | CNUHHM000013 | KY367042 |
| 1-52 | Mirinae | *Adelphocoris* | *piceosetosus* | Korea, GB  35.87N 128.35E | 19-Aug-15 | CNUHHM000014 | KY367043 |
| 1-17 | Mirinae | *Adelphocoris* | *quadripunctatus* | Korea, GG  37.21N 126.64E | 21-Aug-04 | CNUHHM040009 | KY367044 |
| 1-40 | Mirinae | *Adelphocoris* | *quadripunctatus* | Korea, GW  37.71N 128.71E | 11-May-05 | CNUHHM041036 | KY367045 |
| 4-53 | Mirinae | *Adelphocoris* | *quadripunctatus* | Korea, GW  37.71N 128.71E | 11-May-05 | CNUHHM043050 | KY367046 |
| 4-54 | Mirinae | *Adelphocoris* | *quadripunctatus* | Korea, GW  37.71N 128.71E | 12-May-05 | CNUHHM043051 | KY367047 |
| 0-15 | Mirinae | *Adelphocoris* | *reichelii* | Korea, GW  37.71N 128.71E | 17-Jul-15 | CNUHHM037044 | KY367048 |
| 6-1 | Mirinae | *Adelphocoris* | *reichelii* | Korea, JB  35.32N 126.66E | 6-Aug-14 | CNUHHM014021 | KY367049 |
| 0-2 | Mirinae | *Adelphocoris* | *suturalis* | Korea, JJ  33.43N 126.62E | 21-Sep-14 | CNUHHM023001 | KY367050 |
| 0-6 | Mirinae | *Adelphocoris* | *suturalis* | Korea, CN  36.37N 127.35E | 24-Aug-15 | CNUHHM034058 | KY367051 |
| 1-19 | Mirinae | *Adelphocoris* | *suturalis* | Korea, CN  36.37N 127.35E | 26-May-15 | CNUHHM039068 | KY367052 |
| 0-5 | Mirinae | *Adelphocoris* | *triannulatus* | Korea, JJ  33.43N 126.62E | 27-Jun-15 | CNUHHM040037 | KY367053 |
| 1-41 | Mirinae | *Adelphocoris* | *triannulatus* | Korea, CN  36.37N 127.35E | 29-Aug-15 | CNUHHM041030 | KY367054 |
| 3-9 | Mirinae | *Adelphocoris* | *triannulatus* | Korea, GW  37.71N 128.71E | 17-Sep-15 | CNUHHM042020 | KY367055 |
| 4-32 | Mirinae | *Adelphocorisella* | *lespedezae* | Korea, JJ  33.43N 126.62E | 3-Sep-15 | CNUHHM036009 | KY367056 |
| 4-33 | Mirinae | *Adelphocorisella* | *lespedezae* | Korea, CN  36.37N 127.35E | 4-Aug-14 | CNUHHM021031 | KY367057 |
| 3-25 | Mirinae | *Apolygus* | *atriclavus* | Korea, GW  37.71N 128.71E | 19-Sep-15 | CNUHHM000033 | KY367058 |
| 3-26 | Mirinae | *Apolygus* | *atriclavus* | Korea, GW  37.71N 128.71E | 19-Sep-15 | CNUHHM000034 | KY367059 |
| 2-60 | Mirinae | *Apolygus* | *cuneofasciatus* | Korea, GG  37.21N 126.64E | 3-Jul-14 | CNUHHM000030 | KY367060 |
| 3-27 | Mirinae | *Apolygus* | *fraxinicola* | Korea, GW  37.71N 128.71E | 10-Jul-14 | CNUHHM023052 | KY367061 |
| 2-58 | Mirinae | *Apolygus* | *hilaris* | Korea, GG  37.21N 126.64E | 11-Aug-15 | CNUHHM000027 | KY367062 |
| 2-59 | Mirinae | *Apolygus* | *hilaris* | Korea, GG  37.21N 126.64E | 11-Aug-15 | CNUHHM000028 | KY367063 |
| 4-27 | Mirinae | *Apolygus* | *hilaris* | Korea, CB  36.37N 128.46E | 6-Aug-15 | CNUHHM033023 | KY367064 |
| 4-30 | Mirinae | *Apolygus* | *hilaris* | Korea, CN  36.37N 127.35E | 23-Jun-15 | CNUHHM040060 | KY367065 |
| 1-5 | Mirinae | *Apolygus* | *lucorum* | Korea, CN  36.37N 127.35E | 12-Aug-15 | CNUHHM033054 | KY367066 |
| 2-39 | Mirinae | *Apolygus* | *lucorum* | Korea, CN  36.37N 127.35E | 8-Jun-15 | CNUHHM030023 | KY367067 |
| 2-54 | Mirinae | *Apolygus* | *lucorum* | Korea, GG  37.21N 126.64E | 3-Jul-14 | CNUHHM000019 | KY367068 |
| 3-4 | Mirinae | *Apolygus* | *lucorum* | Korea, GG  37.21N 126.64E | 16-May-15 | CNUHHM032053 | KY367069 |
| 3-6 | Mirinae | *Apolygus* | *lucorum* | Korea, GG  37.21N 126.64E | 3-Jul-14 | CNUHHM039040 | KY367070 |
| 1-8 | Mirinae | *Apolygus* | *pulchellus* | Korea, CN  36.37N 127.35E | 24-Aug-15 | CNUHHM034045 | KY367071 |
| 1-9 | Mirinae | *Apolygus* | *pulchellus* | Korea, CN  36.37N 127.35E | 24-Aug-15 | CNUHHM034048 | KY367072 |
| 2-56 | Mirinae | *Apolygus* | *pulchellus* | Korea, GG  37.21N 126.64E | 11-Aug-15 | CNUHHM000026 | KY367073 |
| 0-31 | Mirinae | *Apolygus* | *roseofemoralis* | Korea, JN  34.35N 126.66E | 25-Jul-16 | CNUHHM043018 | KY367074 |
| 1-7 | Mirinae | *Apolygus* | *roseofemoralis* | Korea, JJ  33.43N 126.62E | 3-Sep-15 | CNUHHM036024 | KY367075 |
| 3-2 | Mirinae | *Apolygus* | *roseofemoralis* | Korea, GG  37.21N 126.64E | 11-Aug-15 | CNUHHM000031 | KY367076 |
| 1-3 | Mirinae | *Apolygus* | *spinolae* | Korea, JJ  33.43N 126.62E | 20-Sep-14 | CNUHHM022066 | KY367077 |
| 2-4 | Mirinae | *Apolygus* | *spinolae* | Korea, GN  34.47N 128.69E | 8-May-14 | CNUHHM013082 | KY367078 |
| 2-5 | Mirinae | *Apolygus* | *spinolae* | Korea, CB  36.37N 128.46E | 2-Aug-15 | CNUHHM035034 | KY367079 |
| 2-42 | Mirinae | *Apolygus* | *spinolae* | Korea, JJ  33.43N 126.62E | 20-Sep-14 | CNUHHM040017 | KY367080 |
| 2-43 | Mirinae | *Apolygus* | *spinolae* | Korea, CN  36.37N 127.35E | 27-May-15 | CNUHHM034018 | KY367081 |
| 2-51 | Mirinae | *Apolygus* | *spinolae* | Korea, GW  37.71N 128.71E | 17-Sep-15 | CNUHHM041069 | KY367082 |
| 2-38 | Mirinae | *Apolygus* | *subhilaris* | Korea, GW  37.71N 128.71E | 17-Sep-15 | CNUHHM041072 | KY367083 |
| 2-52 | Mirinae | *Apolygus* | *subhilaris* | Korea, GW  37.71N 128.71E | 29-Jun-16 | CNUHHM042065 | KY367084 |
| 4-31 | Mirinae | *Apolygus* | *subhilaris* | Korea, GW  37.71N 128.71E | 10-Jul-14 | CNUHHM023050 | KY367085 |
| 5-2 | Mirinae | *Apolygus* | *subhilaris* | Korea, GW  37.71N 128.71E | 25-Jul-16 | CNUHHM043053 | KY367086 |
| 0-30 | Mirinae | *Apolygus* | *subpulchellus* | Korea, JN  34.35N 126.66E | 25-Jul-16 | CNUHHM043014 | KY367087 |
| 2-55 | Mirinae | *Apolygus* | *subpulchellus* | Korea, GG  37.21N 126.64E | 11-Aug-15 | CNUHHM000024 | KY367088 |
| 4-25 | Mirinae | *Apolygus* | *subpulchellus* | Korea, GG  37.21N 126.64E | 10-Jul-15 | CNUHHM040006 | KY367089 |
| 2-3 | Mirinae | *Apolygus* | *watajii* | Korea, GG  37.21N 126.64E | 3-Aug-15 | CNUHHM040036 | KY367090 |
| 3-1 | Mirinae | *Apolygus* | *watajii* | Korea, GW  37.71N 128.71E | 17-Jul-15 | CNUHHM037076 | KY367091 |
| 3-29 | Mirinae | *Apolygus* | *watajii* | Korea, GG  37.21N 126.64E | 8-Jun-15 | CNUHHM030030 | KY367092 |
| 2-46 | Mirinae | *Bertsa* | *lankana* | Korea, JJ  33.43N 126.62E | 3-Sep-15 | CNUHHM036015 | KY367093 |
| 2-47 | Mirinae | *Bertsa* | *lankana* | Korea, JJ  33.43N 126.62E | 3-Sep-15 | CNUHHM036016 | KY367094 |
| 5-37 | Mirinae | *Bertsa* | *lankana* | Korea, JJ  33.43N 126.62E | 3-Sep-15 | CNUHHM036026 | KY367095 |
| 5-38 | Mirinae | *Bertsa* | *lankana* | Korea, JJ  33.43N 126.62E | 3-Sep-15 | CNUHHM036027 | KY367096 |
| 0-25 | Mirinae | *Capsodes* | *gothicus* | Korea, GG  37.21N 126.64E | 29-May-14 | CNUHHM000001 | KY367097 |
| 4-55 | Mirinae | *Capsodes* | *gothicus* | Korea, GW  37.71N 128.71E | 25-Jun-16 | CNUHHM000056 | KY367098 |
| 0-19 | Mirinae | *Capsus* | *koreanus* | Korea, CN  36.37N 127.35E | 11-May-15 | CNUHHM041005 | KY367099 |
| 3-14 | Mirinae | *Capsus* | *koreanus* | Korea, CN  36.37N 127.35E | 24-May-15 | CNUHHM039011 | KY367100 |
| 3-15 | Mirinae | *Capsus* | *koreanus* | Korea, GG  37.21N 126.64E | 16-May-15 | CNUHHM032065 | KY367101 |
| 4-56 | Mirinae | *Capsus* | *pilifer* | Korea, GW  37.71N 128.71E | 30-Jun-16 | CNUHHM000057 | KY367102 |
| 0-13 | Mirinae | *Castanopsides* | *kerzhneri* | Korea, CN  36.37N 127.35E | 7-May-14 | CNUHHM012046 | KY367103 |
| 0-16 | Mirinae | *Castanopsides* | *kerzhneri* | Korea, CN  36.37N 127.35E | 5-May-15 | CNUHHM027056 | KY367104 |
| 2-13 | Mirinae | *Castanopsides* | *kerzhneri* | Korea, GW  37.71N 128.71E | 28-May-15 | CNUHHM030004 | KY367105 |
| 0-14 | Mirinae | *Castanopsides* | *potanini* | Korea, CN  36.37N 127.35E | 26-May-14 | CNUHHM002049 | KY367106 |
| 0-17 | Mirinae | *Castanopsides* | *potanini* | Korea, CB  36.37N 128.46E | 18-Jun-15 | CNUHHM042001 | KY367107 |
| 2-14 | Mirinae | *Castanopsides* | *potanini* | Korea, GW  37.71N 128.71E | 28-May-15 | CNUHHM030003 | KY367108 |
| 0-63 | Mirinae | *Charagochilus* | *angusticollis* | Korea, JN  34.35N 126.66E | 25-Jul-16 | CNUHHM043024 | KY367109 |
| 2-26 | Mirinae | *Charagochilus* | *angusticollis* | Korea, CN  36.37N 127.35E | 9-May-14 | CNUHHM026017 | KY367110 |
| 2-8 | Mirinae | *Creontiades* | *coloripes* | Korea, CN  36.37N 127.35E | 27-Jul-15 | CNUHHM038033 | KY367111 |
| 5-13 | Mirinae | *Creontiades* | *coloripes* | Korea, JJ  33.43N 126.62E | 2-Sep-15 | CNUHHM035059 | KY367112 |
| 5-14 | Mirinae | *Creontiades* | *coloripes* | Korea, JJ  33.43N 126.62E | 2-Sep-15 | CNUHHM035060 | KY367113 |
| 5-15 | Mirinae | *Creontiades* | *coloripes* | Korea, JJ  33.43N 126.62E | 2-Sep-15 | CNUHHM035058 | KY367114 |
| 1-31 | Mirinae | *Cyphodemidea* | *saundersi* | Korea, GW  37.71N 128.71E | 29-May-15 | CNUHHM030018 | KY367115 |
| 5-24 | Mirinae | *Cyphodemidea* | *saundersi* | Korea, GW  37.71N 128.71E | 24-Apr-14 | CNUHHM024025 | KY367116 |
| 5-25 | Mirinae | *Cyphodemidea* | *saundersi* | Korea, GW  37.71N 128.71E | 24-Apr-14 | CNUHHM024026 | KY367117 |
| 0-4 | Mirinae | *Eurystylus* | *coelestialium* | Korea, CN  36.37N 127.35E | 12-Aug-14 | CNUHHM008062 | KY367118 |
| 0-29 | Mirinae | *Eurystylus* | *coelestialium* | Korea, GW  37.71N 128.71E | 16-Jul-15 | CNUHHM035010 | KY367119 |
| 0-1 | Mirinae | *Eurystylus* | *luteus* | Korea, JJ  33.43N 126.62E | 3-Sep-15 | CNUHHM036011 | KY367120 |
| 0-3 | Mirinae | *Eurystylus* | *luteus* | Korea, CN  36.37N 127.35E | 30-Jul-14 | CNUHHM003066 | KY367121 |
| 0-28 | Mirinae | *Eurystylus* | *luteus* | Korea, CN  36.37N 127.35E | 28-Jul-14 | CNUHHM015019 | KY367122 |
| 2-17 | Mirinae | *Josifovolygus* | *niger* | Korea, GG  37.21N 126.64E | 16-May-15 | CNUHHM038062 | KY367123 |
| 2-18 | Mirinae | *Josifovolygus* | *niger* | Korea, GG  37.21N 126.64E | 16-May-15 | CNUHHM038075 | KY367124 |
| 5-30 | Mirinae | *Josifovolygus* | *niger* | Korea, GW  37.71N 128.71E | 28-May-15 | CNUHHM030002 | KY367125 |
| 2-7 | Mirinae | *Koreocoris* | *bicoloratus* | Korea, JJ  33.43N 126.62E | 19-Jun-14 | CNUHHM024077 | KY367126 |
| 5-17 | Mirinae | *Koreocoris* | *bicoloratus* | Korea, CN  36.37N 127.35E | 13-Jun-14 | CNUHHM011051 | KY367127 |
| 5-18 | Mirinae | *Koreocoris* | *bicoloratus* | Korea, CN  36.37N 127.35E | 13-Jun-14 | CNUHHM011054 | KY367128 |
| 0-26 | Mirinae | *Loristes* | *decoratus* | Korea, CN  36.37N 127.35 | 12-May-14 | CNUHHM015069 | KY367129 |
| 2-9 | Mirinae | *Loristes* | *decoratus* | Korea, GG  37.21N 126.64E | 16-May-15 | CNUHHM034032 | KY367130 |
| 5-19 | Mirinae | *Loristes* | *decoratus* | Korea, GG  37.21N 126.64E | 16-May-15 | CNUHHM032038 | KY367131 |
| 5-20 | Mirinae | *Loristes* | *decoratus* | Korea, JN  34.35N 126.66E | 2-May-15 | CNUHHM027052 | KY367132 |
| 0-22 | Mirinae | *Lygocoris* | *pabulinus* | Korea, JB  35.32N 126.66E | 31-Jul-14 | CNUHHM025015 | KY367133 |
| 2-49 | Mirinae | *Lygocoris* | *pabulinus* | Korea, GW  37.71N 128.71E | 29-Jun-16 | CNUHHM042067 | KY367134 |
| 5-1 | Mirinae | *Lygocoris* | *pabulinus* | Korea, GW  37.71N 128.71E | 25-Jul-16 | CNUHHM043055 | KY367135 |
| 0-23 | Mirinae | *Lygus* | *rugulipennis* | Korea, GW  37.71N 128.71E | 17-Jul-15 | CNUHHM034077 | KY367136 |
| 0-24 | Mirinae | *Lygus* | *rugulipennis* | Korea, GW  37.71N 128.71E | 7-Aug-15 | CNUHHM041051 | KY367137 |
| 2-10 | Mirinae | *Mermitelocerus* | *annulipes* | Korea, GW  37.71N 128.71E | 6-Jun-15 | CNUHHM030082 | KY367138 |
| 5-27 | Mirinae | *Mermitelocerus* | *annulipes* | Korea, GW  37.71N 128.71E | 6-Jun-15 | CNUHHM030081 | KY367139 |
| 4-28 | Mirinae | gen. nov. |  | Korea, GW  37.71N 128.71E | 17-Jul-15 | CNUHHM037050 | KY367140 |
| 4-29 | Mirinae | gen. nov. |  | Korea, GG  37.21N 126.64E | 3-Jul-14 | CNUHHM039036 | KY367141 |
| 0-20 | Mirinae | *Neolygus* | *tiliicola* | Korea, GW  37.71N 128.71E | 17-Aug-15 | CNUHHM041071 | KY367142 |
| 5-4 | Mirinae | *Neolygus* | *tiliicola* | Korea, GW  37.71N 128.71E | 25-Jul-16 | CNUHHM043056 | KY367143 |
| 2-20 | Mirinae | *Orthops* | *scutellatus* | Korea, GW  37.71N 128.71E | 16-Jul-15 | CNUHHM037004 | KY367144 |
| 2-21 | Mirinae | *Orthops* | *scutellatus* | Korea, CN  36.37N 127.35E | 14-Jul-15 | CNUHHM040043 | KY367145 |
| 5-46 | Mirinae | *Orthops* | *scutellatus* | Korea, CB  36.37N 128.46E | 2-Aug-15 | CNUHHM035037 | KY367146 |
| 5-47 | Mirinae | *Orthops* | *scutellatus* | Korea, GW  37.71N 128.71E | 20-Jul-16 | CNUHHM042043 | KY367147 |
| 2-24 | Mirinae | *Philostephanus* | *glaber* | Korea, CN  36.37N 127.35E | 6-Jun-15 | CNUHHM031009 | KY367148 |
| 4-58 | Mirinae | *Philostephanus* | *rubripes* | Korea, CB  36.37N 128.46E | 18-Jun-15 | CNUHHM039071 | KY367149 |
| 4-60 | Mirinae | *Philostephanus* | *ulmi* | Korea, GW  37.71N 128.71E | 29-Jun-16 | CNUHHM042073 | KY367150 |
| 3-19 | Mirinae | *Phytocoris* | *longipennis* | Korea, CN  36.37N 127.35E | 1-Jun-15 | CNUHHM029025 | KY367151 |
| 3-20 | Mirinae | *Phytocoris* | *longipennis* | Korea, CB  36.37N 128.46E | 18-Jun-15 | CNUHHM041016 | KY367152 |
| 4-48 | Mirinae | *Phytocoris* | *longipennis* | Korea, GW  37.71N 128.71E | 17-Jul-15 | CNUHHM037052 | KY367153 |
| 0-27 | Mirinae | *Phytocoris* | *shabliovskii* | Korea, CN  36.37N 127.35E | 28-Jul-14 | CNUHHM015044 | KY367154 |
| 4-45 | Mirinae | *Phytocoris* | *shabliovskii* | Korea, GW  37.71N 128.71E | 17-Jul-15 | CNUHHM037053 | KY367155 |
| 4-51 | Mirinae | *Pinalitus* | *nigriceps* | Korea, GW  37.71N 128.71E | 5-Jul-14 | CNUHHM022035 | KY367156 |
| 4-52 | Mirinae | *Pinalitus* | *nigriceps* | Korea, GW  37.71N 128.71E | 29-Jun-16 | CNUHHM042081 | KY367157 |
| 5-36 | Mirinae | *Pinalitus* | *nigriceps* | Korea, GW  37.71N 128.71E | 30-Jun-16 | CNUHHM043065 | KY367158 |
| 4-49 | Mirinae | *Pinalitus* | *lubeolus* | Korea, GG  37.21N 126.64E | 21-Jul-16 | CNUHHM042056 | KY367159 |
| 4-50 | Mirinae | *Pinalitus* | *lubeolus* | Korea, GW  37.71N 128.71E | 29-Jun-16 | CNUHHM042079 | KY367160 |
| 5-35 | Mirinae | *Pinalitus* | *lubeolus* | Korea, GW  37.71N 128.71E | 29-Jun-16 | CNUHHM042080 | KY367161 |
| 1-35 | Mirinae | *Polymerias* | *opacipennis* | Korea, GW  37.71N 128.71E | 29-May-15 | CNUHHM030014 | KY367162 |
| 1-36 | Mirinae | *Polymerias* | *opacipennis* | Korea, JN  34.35N 126.66E | 2-May-15 | CNUHHM027029 | KY367163 |
| 5-28 | Mirinae | *Polymerias* | *opacipennis* | Korea, GG  37.21N 126.64E | 16-May-15 | CNUHHM038063 | KY367164 |
| 5-29 | Mirinae | *Polymerias* | *opacipennis* | Korea, GG  37.21N 126.64E | 16-May-15 | CNUHHM038073 | KY367165 |
| 2-30 | Mirinae | *Polymerus* | *amurensis* | Korea, CN  36.37N 127.35E | 24-May-15 | CNUHHM039003 | KY367166 |
| 0-56 | Mirinae | *Polymerus* | *cognatus* | Korea, GG  37.21N 126.64E | 4-Jul-14 | CNUHHM010051 | KY367167 |
| 1-37 | Mirinae | *Polymerus* | *cognatus* | Korea, CN  36.37N 127.35E | 31-Jul-15 | CNUHHM038004 | KY367168 |
| 2-27 | Mirinae | *Polymerus* | *cognatus* | Korea, CB  36.37N 128.46E | 2-Aug-15 | CNUHHM035030 | KY367169 |
| 2-29 | Mirinae | *Polymerus* | *cognatus* | Korea, GW  37.71N 128.71E | 31-Aug-15 | CNUHHM041060 | KY367170 |
| 2-28 | Mirinae | *Polymerus* | *pekinensis* | Korea, CN  36.37N 127.35E | 26-May-15 | CNUHHM039065 | KY367171 |
| 5-58 | Mirinae | *Polymerus* | *pekinensis* | Korea, GG  37.21N 126.64E | 21-Jul-16 | CNUHHM042057 | KY367172 |
| 0-64 | Mirinae | *Proboscidocoris* | *varicornis* | Korea, JN  34.35N 126.66E | 21-Jun-08 | CNUHHM027016 | KY367173 |
| 1-39 | Mirinae | *Proboscidocoris* | *varicornis* | Korea, CB  36.37N 128.46E | 21-Jun-08 | CNUHHM035031 | KY367174 |
| 3-38 | Mirinae | *Proboscidocoris* | *varicornis* | Korea, CN  36.37N 127.35E | 20-May-08 | CNUHHM011018 | KY367175 |
| 2-36 | Mirinae | *Rhabdomiris* | sp. nov. | Korea, GW  37.71N 128.55E | 29-May-15 | CNUHHM040055 | KY229060 |
| 4-17 | Mirinae | *Rhabdomiris* | sp. nov. | Korea, GW  37.71N 128.55E | 29-May-15 | CNUHHM040053 | KY229061 |
| 2-35 | Mirinae | *Rhabdomiris* | *pulcherrimus* | Korea, GG  37.58N 127.45E | 16-May-15 | CNUHHM034034 | KY229058 |
| 2-41 | Mirinae | *Rhabdomiris* | *pulcherrimus* | Korea, GW  37.71N 128.55E | 29-May-15 | CNUHHM040055 | KY229059 |
| 0-32 | Mirinae | *Stenodema* | *rubrinervis* | Korea, CN  36.37N 127.35E | 25-Apr-14 | CNUHHM026079 | KY367176 |
| 1-43 | Mirinae | *Stenodema* | *rubrinervis* | Korea, CN  36.37N 127.35E | 8-Sep-15 | CNUHHM041024 | KY367177 |
| 1-44 | Mirinae | *Stenodema* | *rubrinervis* | Korea, JN  34.35N 126.66E | 25-Jul-16 | CNUHHM043030 | KY367178 |
| 0-33 | Mirinae | *Stenodema* | *sibirica* | Korea, GW  37.71N 128.71E | 16-Jul-15 | CNUHHM034068 | KY367179 |
| 0-34 | Mirinae | *Stenodema* | *sibirica* | Korea, GW  37.71N 128.71E | 9-Jul-14 | CNUHHM008012 | KY367180 |
| 2-11 | Mirinae | *Stenotus* | *binotatus* | Korea, GG  37.21N 126.64E | 9-Jun-15 | CNUHHM030040 | KY367181 |
| 5-31 | Mirinae | *Stenotus* | *binotatus* | Korea, GW  37.71N 128.71E | 29-Jun-16 | CNUHHM042072 | KY367182 |
| 5-32 | Mirinae | *Stenotus* | *binotatus* | Korea, GW  37.71N 128.71E | 29-Jun-16 | CNUHHM000061 | KY367183 |
| 0-18 | Mirinae | *Stenotus* | *rubrovittatus* | Korea, CB  36.37N 128.46E | 6-Aug-15 | CNUHHM033044 | KY367184 |
| 2-12 | Mirinae | *Stenotus* | *rubrovittatus* | Korea, CN  36.37N 127.35E | 10-Jun-15 | CNUHHM030066 | KY367185 |
| 5-33 | Mirinae | *Stenotus* | *rubrovittatus* | Korea, CN  36.37N 127.35E | 22-Jul-15 | CNUHHM040081 | KY367186 |
| 5-34 | Mirinae | *Stenotus* | *rubrovittatus* | Korea, JJ  33.43N 126.62E | 2-Sep-15 | CNUHHM035063 | KY367187 |
| 0-21 | Mirinae | *Taylorilygus* | *apicalis* | Korea, JN  34.35N 126.66E | 25-Jul-16 | CNUHHM043019 | KY367188 |
| 3-23 | Mirinae | *Taylorilygus* | *apicalis* | Korea, JJ  33.43N 126.62E | 21-Sep-14 | CNUHHM023003 | KY367189 |
| 3-24 | Mirinae | *Taylorilygus* | *apicalis* | Korea, JJ  33.43N 126.62E | 22-Sep-14 | CNUHHM024034 | KY367190 |
| 3-21 | Mirinae | *Tinginotum* | *perlatum* | Korea, JJ  33.43N 126.62E | 5-Oct-15 | CNUHHM041053 | KY367191 |
| 3-22 | Mirinae | *Tinginotum* | *perlatum* | Korea, JJ  33.43N 126.62E | 3-Sep-15 | CNUHHM036028 | KY367192 |
| 5-39 | Mirinae | *Tinginotum* | *perlatum* | Korea, JJ  33.43N 126.62E | 18-Jun-14 | CNUHHM039075 | KY367193 |
| 5-40 | Mirinae | *Tinginotum* | *perlatum* | Korea, JJ  33.43N 126.62E | 18-Jun-14 | CNUHHM039076 | KY367194 |
| 3-36 | Mirinae | *Tinginotum* | *pini* | Korea, GW  37.71N 128.71E | 1-Sep-15 | CNUHHM041026 | KY367195 |
| 4-2 | Orthotylinae | *Cyllecoris* | *nakanishii* | Korea, GW  37.71N 128.71E | 28-May-15 | CNUHHM030009 | KY367196 |
| 0-73 | Orthotylinae | *Cyrtorhinus* | *lividipennis* | Korea, JN  34.35N 126.66E | 29-Sep-16 | CNUHHM000007 | KY367197 |
| 0-74 | Orthotylinae | *Cyrtorhinus* | *lividipennis* | Korea, JN  34.35N 126.66E | 29-Sep-16 | CNUHHM000008 | KY367198 |
| 0-65 | Orthotylinae | *Dryophilocoris* | *kerzhneri* | Korea, CN  36.37N 127.35E | 25-Apr-14 | CNUHHM026049 | KY367199 |
| 0-68 | Orthotylinae | *Dryophilocoris* | *miyamotoi* | Korea, CN  36.37N 127.35E | 23-Apr-15 | CNUHHM029021 | KY367200 |
| 0-35 | Orthotylinae | *Ectmetopterus* | *comitans* | Korea, JN  34.35N 126.66E | 18-Aug-15 | CNUHHM038050 | KY367201 |
| 4-6 | Orthotylinae | *Ectmetopterus* | *comitans* | Korea, CN  36.37N 127.35E | 7-Aug-14 | CNUHHM015063 | KY367202 |
| 4-7 | Orthotylinae | *Ectmetopterus* | *micantulus* | Korea, JB  35.32N 126.66E | 31-Jul-14 | CNUHHM025036 | KY367203 |
| 4-8 | Orthotylinae | *Ectmetopterus* | *micantulus* | Korea, JN  34.35N 126.66E | 25-Jul-16 | CNUHHM043041 | KY367204 |
| 0-71 | Orthotylinae | *Orthocephalus* | *funestus* | Korea, CN  36.37N 127.35E | 11-Jun-14 | CNUHHM022007 | KY367205 |
| 0-69 | Orthotylinae | *Orthotylus* | *flavosparsus* | Korea, CN  36.37N 127.35E | 1-Jun-15 | CNUHHM029046 | KY367206 |
| 3-31 | Orthotylinae | *Orthotylus* | *flavosparsus* | Korea, GW  37.71N 128.71E | 20-Jul-16 | CNUHHM042031 | KY367207 |
| 0-66 | Orthotylinae | *Orthotylus* | *interpositus* | Korea, GW  37.71N 128.71E | 30-Jun-16 | CNUHHM043068 | KY367208 |
| 5-7 | Orthotylinae | *Orthotylus* | *interpositus* | Korea, GW  37.71N 128.71E | 30-Jun-16 | CNUHHM043071 | KY367209 |
| 5-8 | Orthotylinae | *Orthotylus* | *interpositus* | Korea, GW  37.71N 128.71E | 30-Jun-16 | CNUHHM043072 | KY367210 |
| 0-70 | Orthotylinae | *Orthotylus* | *pallens* | Korea, CN  36.37N 127.35E | 19-May-15 | CNUHHM034003 | KY367211 |
| 4-9 | Orthotylinae | *Orthotylus* | *pallens* | Korea, CN  36.37N 127.35E | 19-May-15 | CNUHHM034002 | KY367212 |
| 5-9 | Orthotylinae | *Orthotylus* | *pallens* | Korea, GW  37.71N 128.71E | 30-Jun-16 | CNUHHM043067 | KY367213 |
| 3-45 | Orthotylinae | *Orthotylus* | *salicis* | Korea, GW  37.71N 128.71E | 9-Jul-14 | CNUHHM008010 | KY367214 |
| 5-59 | Orthotylinae | *Orthotylus* | *salicis* | Korea, GW  37.71N 128.71E | 10-Jul-14 | CNUHHM015007 | KY367215 |
| 4-10 | Orthotylinae | *Orthotylus* | *sophorae* | Korea, CN  36.37N 127.35E | 27-May-15 | CNUHHM034024 | KY367216 |
| 5-10 | Orthotylinae | *Orthotylus* | *sophorae* | Korea, GW  37.71N 128.71E | 30-Jun-16 | CNUHHM043069 | KY367217 |
| 5-11 | Orthotylinae | *Orthotylus* | *sophorae* | Korea, GW  37.71N 128.71E | 30-Jun-16 | CNUHHM043070 | KY367218 |
| 4-5 | Orthotylinae | *Pseudoloxops* | *miyamotoi* | Korea, CN  36.37N 127.35E | 31-Jul-15 | CNUHHM038014 | KY367219 |
| 4-4 | Orthotylinae | *Pseudoloxops* | *miyatakei* | Korea, JB  35.32N 126.66E | 4-Aug-14 | CNUHHM036060 | KY367220 |
| 2-44 | Orthotylinae | *Zanchius* | *tarasovi* | Korea, CB  36.37N 128.46E | 6-Aug-15 | CNUHHM033010 | KY367221 |
| 2-45 | Orthotylinae | *Zanchius* | *tarasovi* | Korea, CN  36.37N 127.35E | 16-Sep-14 | CNUHHM024036 | KY367222 |
| 0-49 | Phylinae | *Acrorrhinium* | *inexpectatum* | Korea, CB  36.37N 128.46E | 6-Aug-15 | CNUHHM033046 | KY367223 |
| 3-34 | Phylinae | *Acrorrhinium* | *inexpectatum* | Korea, GG  37.21N 126.64E | 11-Aug-15 | CNUHHM000047 | KY367224 |
| 5-41 | Phylinae | *Acrorrhinium* | *inexpectatum* | Korea, CN  36.37N 127.35E | 10-Sep-14 | CNUHHM040029 | KY367225 |
| 0-62 | Phylinae | *Campylomma* | *annulicorne* | Korea, JJ  33.43N 126.62E | 27-Jun-15 | CNUHHM040041 | KY367226 |
| 0-50 | Phylinae | *Compsidolon* | *salicellum* | Korea, CN  36.37N 127.35E | 28-Jul-14 | CNUHHM015030 | KY367227 |
| 0-51 | Phylinae | *Europiella* | *artemisiae* | Korea, GW  37.71N 128.71E | 29-Jun-16 | CNUHHM043006 | KY367228 |
| 0-58 | Phylinae | *Europiella* | *kiritshenkoi* | Korea, GW  37.71N 128.71E | 17-Jul-15 | CNUHHM037059 | KY367229 |
| 4-21 | Phylinae | *Hallodapus* | *linnavouri* | Korea, JJ  33.43N 126.62E | 3-Sep-15 | CNUHHM036021 | KY367230 |
| 0-59 | Phylinae | *Harpocera* | *choii* | Korea, CN  36.37N 127.35E | 18-Apr-14 | CNUHHM015068 | KY367231 |
| 1-28 | Phylinae | *Harpocera* | *choii* | Korea, GW  37.71N 128.71E | 29-May-15 | CNUHHM000011 | KY367232 |
| 2-31 | Phylinae | *Harpocera* | *choii* | Korea, GW  37.71N 128.71E | 29-May-15 | CNUHHM000015 | KY367233 |
| 1-29 | Phylinae | *Harpocera* | *josifovi* | Korea, GW  37.71N 128.71E | 29-May-15 | CNUHHM000012 | KY367234 |
| 2-32 | Phylinae | *Harpocera* | *josifovi* | Korea, GW  37.71N 128.71E | 29-May-15 | CNUHHM000016 | KY367235 |
| 2-33 | Phylinae | *Harpocera* | *josifovi* | Korea, GW  37.71N 128.71E | 29-May-15 | CNUHHM000017 | KY367236 |
| 2-34 | Phylinae | *Harpocera* | *koreana* | Korea, CN  36.37N 127.35E | 18-Apr-14 | CNUHHM015066 | KY367237 |
| 5-21 | Phylinae | *Harpocera* | *koreana* | Korea, JN  34.35N 126.66E | 1-May-15 | CNUHHM027010 | KY367238 |
| 5-22 | Phylinae | *Harpocera* | *koreana* | Korea, JN  34.35N 126.66E | 1-May-15 | CNUHHM027011 | KY367239 |
| 5-23 | Phylinae | *Harpocera* | *koreana* | Korea, CN  36.37N 127.35E | 5-May-15 | CNUHHM027048 | KY367240 |
| 0-55 | Phylinae | *Phylus* | *coryloides* | Korea, CN  36.37N 127.35E | 19-May-14 | CNUHHM004052 | KY367241 |
| 0-45 | Phylinae | *Pilophorus* | *clavatus* | Korea, JN  34.35N 126.66E | 25-Jul-16 | CNUHHM043035 | KY367242 |
| 4-20 | Phylinae | *Pilophorus* | *niger* | Korea, CN  36.37N 127.35E | 27-May-15 | CNUHHM034031 | KY367243 |
| 4-14 | Phylinae | *Pilophorus* | *okamotoi* | Korea, CN  36.37N 127.35E | 23-Jun-15 | CNUHHM040076 | KY367244 |
| 4-15 | Phylinae | *Pilophorus* | *setulosus* | Korea, CN  36.37N 127.35E | 23-Jun-15 | CNUHHM040075 | KY367245 |
| 0-44 | Phylinae | *Pilophorus* | *typicus* | Korea, JJ  33.43N 126.62E | 5-Sep-15 | CNUHHM036005 | KY367246 |
| 4-19 | Phylinae | *Pilophorus* | *typicus* | Korea, GG  37.21N 126.64E | 10-Jul-15 | CNUHHM040013 | KY367247 |
| 0-46 | Phylinae | *Plagiognathus* | *amurensis* | Korea, CN  36.37N 127.35E | 14-May-14 | CNUHHM026003 | KY367248 |
| 0-47 | Phylinae | *Plagiognathus* | *amurensis* | Korea, GG  37.21N 126.64E | 3-Jul-14 | CNUHHM039028 | KY367249 |
| 0-48 | Phylinae | *Plagiognathus* | *chrysanthemi* | Korea, GW  37.71N 128.71E | 17-Jul-15 | CNUHHM037035 | KY367250 |
| 0-57 | Phylinae | *Plagiognathus* | *collaris* | Korea, GW  37.71N 128.71E | 29-Jun-16 | CNUHHM043005 | KY367251 |
| 0-53 | Phylinae | *Psallus* | *atratus* | Korea, GW  37.71N 128.71E | 28-May-15 | CNUHHM030007 | KY367252 |
| 0-54 | Phylinae | *Psallus* | *castanea* | Korea, CB  36.37N 128.46E | 18-Jun-15 | CNUHHM039060 | KY367253 |
| 0-60 | Phylinae | *Psallus* | *roseoguttatus* | Korea, GW  37.71N 128.71E | 29-Jun-16 | CNUHHM000004 | KY367254 |
| 4-18 | Phylinae | *Sejanus* | *potanini* | Korea, JJ  33.43N 126.62E | 27-Jun-15 | CNUHHM040039 | KY367255 |
| 0-52 | Phylinae | *Tytthus* | *chinensis* | Korea, GW  37.71N 128.71E | 16-Sep-15 | CNUHHM000003 | KY367256 |
| 0-61 | Phylinae | *Tytthus* | *chinensis* | Korea, GW  37.71N 128.71E | 16-Sep-15 | CNUHHM000005 | KY367257 |
