# Supplementary figures and images for: COI barcoding of plant bugs (Insecta: Hemiptera: Miridae)
Source: PeerJ. 2018 Dec 4;6:e6070. doi: 10.7717/peerj.6070 (PMC6284446; doi:10.7717/peerj.6070)

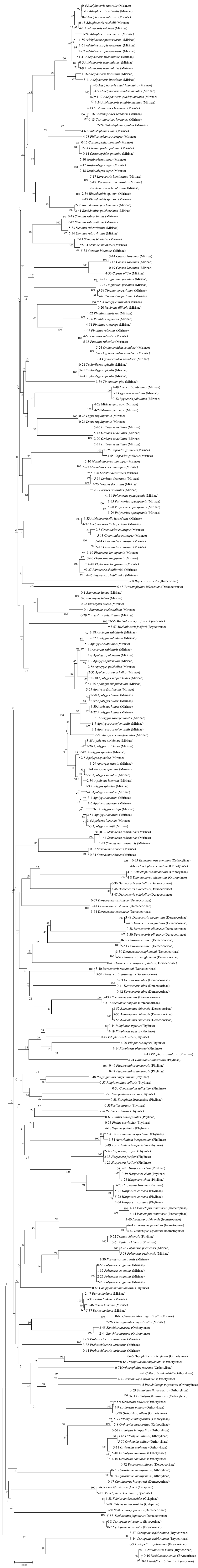

0.02

Supplement: Figure S1 — Parenthesis indicates the subfamily of the species. Numbers indicates specimen numbers corresponding to Table S1. [file peerj-06-6070-s003.pdf]
